# Lysine Biosynthesis Defines a Metabolic Checkpoint for Gibberellin‐Mediated Growth in *Arabidopsis thaliana*

**DOI:** 10.1111/pce.70525

**Published:** 2026-04-16

**Authors:** Débora Gonçalves Gouveia, Wesley E Bhering Barrios, Auxiliadora Oliveira Martins, João Henrique Cavalcanti, Laíse Rosado‐Souza, João Antonio Siqueira, Danilo de Menezes Daloso, Alisdair R. Fernie, Adriano Nunes‐Nesi, Andreas P. M. Weber, Wagner L. Araújo

**Affiliations:** ^1^ National Institute of Science and Technology on Plant Physiology Under Stress Conditions, Departamento de Biologia Vegetal Universidade Federal de Viçosa Viçosa Minas Gerais Brazil; ^2^ Instituto de Educação, Agricultura e Ambiente Universidade Federal do Amazonas Humaitá Amazonas Brazil; ^3^ Max‐Planck‐Institut für Molekulare Pflanzenphysiologie Potsdam‐Golm Germany; ^4^ Departamento de Bioquímica e Biologia Molecular Universidade Federal do Ceará Fortaleza Ceará Brazil; ^5^ Institute of Plant Biochemistry, Cluster of Excellence on Plant Science (CEPLAS) Heinrich‐Heine University Düsseldorf Düsseldorf Germany

**Keywords:** amino acids, gibberellin, L‐diaminopimelate aminotransferase, lysine biosynthesis, metabolic reprogramming

## Abstract

Lysine represents a metabolic hub linking mitochondrial energy status with amino acid catabolism, yet its role in coordinating plant growth and development remains poorly characterised. The *dapat* mutant, which is deficient in *L,L*‐diaminopimelate aminotransferase (DAPAT), exhibits severe growth inhibition associated with broad metabolic imbalance. This metabolic reprogramming is characterised by simultaneous depletion of carbohydrates (sugars and starch), and organic acids, accompanied by elevated amino acid pools, reflecting a profound disruption of carbon–nitrogen balance. These metabolic constraints coincide with altered expression of gibberellin (GA) biosynthetic genes (*CPS*, *KS*, *GA3ox1*), suggesting a potential link between lysine metabolism, energy status, and GA‐mediated growth regulation. We therefore hypothesised that aberrant developmental defects in *dapat* arise from impaired GA metabolism. To test this hypothesis, we treated mutant plants with exogenous GA₃. Multivariate metabolomic analysis revealed a striking disconnect: GA restored the normal morphological phenotype of *dapat* but failed to correct the underlying metabolic dysfunction. GA‐treated *dapat* plants maintain amino acid remobilisation despite growth restoration, as indicated by pronounced *DIN6* induction, a marker of catabolic stress following energy starvation. These findings establish that lysine biosynthesis represents a critical metabolic constraint on plant development, and that growth hormone signalling can decouple morphological rescue from metabolic homoeostasis. This work exposes a fundamental mechanistic uncoupling between hormone‐induced growth and energy balance in lysine‐deficient plants, highlighting the critical role of lysine metabolism in integrating developmental and energetic cues.

## Summary statement

- Lysine biosynthesis integrates carbon–nitrogen balance with gibberellin to control plant growth.
- In an *Arabidopsis* mutant deficient in lysine biosynthesis (*dapat*), gibberellin restores morphology but not metabolism, revealing a decoupling between hormone‐driven growth and energy homoeostasis.

## 1 Introduction

Amino acids, the essential building blocks for proteins, play crucial roles in plant metabolism despite their relatively lower abundance in plants compared to animals due to the predominantly carbohydrate‐based plant structure (Araujo et al. 2011). These compounds serve as vital intermediates in various pathways governing growth and development, while simultaneously participating in signalling cascades that modulate hormonal balance (Häusler et al. 2014; Heinemann and Hildebrandt 2021). Furthermore, extensive research has demonstrated that amino acid catabolism and biosynthesis are fundamental in shaping plant responses to adverse conditions (Galili et al. 2011; Arruda and Barreto 2020; Batista‐Silva et al. 2019; Hildebrandt 2018; Heinemann and Hildebrandt 2021; Yang et al. 2020; Moormann et al. 2022). Yet, amino acid contribution to energy balance during plant development is multifactorial and remains to be fully understood. The catabolism of branched‐chain amino acids (BCAAs) and lysine feeds into an alternative mitochondrial respiratory pathway that utilises amino acids as substrates instead of carbohydrates (Araújo et al. 2011; Izumi et al. 2013). The by‐products of this process sustain respiratory activity under energy starvation conditions through the electron‐transfer flavoprotein: ubiquinone oxidoreductase (ETFQO) and the electron‐transfer flavoprotein (ETF) system (Beckmann and Frerman 1985; Ruzicka and Beinert 1977; Araújo et al. 2010; Araújo et al. 2011; Schertl and Braun 2014; Barros et al. 2017; Engqvist et al. 2009; Hildebrandt et al. 2015; Izumi et al. 2013; Pedrotti et al. 2018).

Among catabolizable amino acids, lysine plays a particular pivotal role as a metabolic bridge linking amino acid degradation to mitochondrial ATP production, thereby connecting amino acid metabolism to the tricarboxylic acid (TCA) cycle and mitochondrial electron transport chain (mETC) (Araújo et al. 2010, 2011). Due to this central function, lysine concentration must be tightly regulated through coordinated control of its biosynthesis and degradation (Galili et al. 2016; Hildebrandt et al. 2015, 2018). In contrast to bacteria, plants employ a more streamlined mechanism for lysine biosynthesis, bypassing multiple steps of *L,L*‐diaminopimelate (*L,L*‐DAP) formation by employing a single enzymatic step catalysed by the enzyme *L,L*‐DAP aminotransferase (*L,L*‐DAPAT) (Hudson et al. 2005; Hudson et al. 2006; Watanabe et al. 2007). Mutations in the *DAPAT* gene resulted in a substantial loss of enzyme activity, triggering not only growth inhibition but also metabolic shifts that resemble a stressed plant phenotype (Cavalcanti et al. 2018). Additionally, microarray analysis of the *dapat* mutant revealed significant alterations in the expression patterns of genes associated with gibberellin (GA) metabolism, raising the possibility that perturbations in lysine content may modulated GA metabolic pathways. This disruption is further associated with imbalances in carbohydrate metabolism and energy homoeostasis (Cavalcanti et al. 2018). While recent studies highlight emerging connections between GA metabolism and amino acid homoeostasis in plants, the molecular mechanism connecting the DAPAT mutation to energy limitation and hormonal regulation remain poorly characterised.

GA biosynthesis is initiated in plastids and completed through sequential enzymatic steps in the endoplasmic reticulum and cytosol (Hedden and Kamiya 1997; Hedden and Thomas 2012, 2016; Hedden 2020). As a key hormonal integrator, bioactive GAs regulate multiple developmental processes – including vegetative and reproductive growth, leaf and root development, and flower and fruit production (Hauvermale et al. 2012; Olszewski et al. 2002; Richards et al. 2001; Ueguchi‐Tanaka et al. 2007; Hernández‐García et al. 2021; Zhang et al. 2023), while remaining tightly coupled to primary metabolism balance (Ribeiro et al. 2012; Paparelli et al. 2013; Araújo et al. 2014; Gasparini et al. 2019; Wu et al. 2021; Zhou et al. 2024).

Energy limitation, often reflected by carbohydrate scarcity, disrupts starch metabolism and consequently suppresses growth through transcriptional repression of *ent*‐kaurene synthase (KS), which is critical for GA biosynthesis (Izumi et al. 2013; Smith and Stitt 2007; Gibon et al. 2009; Sulpice et al. 2009; Stitt and Zeeman 2012). This regulatory coupling is exemplified in starch‐deficient mutants, which show reduced nocturnal growth and constitutively repressed *KS* expression during sugar starvation. These observations establish a regulatory principle wherein energy status controls GA biosynthesis, raising the hypothesis that the profound growth inhibitions in the lysine‐deficient *dapat* mutant may, at least partially, result from altered GA biosynthesis pathway.

To test this hypothesis, we assessed whether exogenous GA₃ could rescue the growth constraints imposed by the DAPAT mutation and analyzed the physiological, metabolic, and molecular responses during vegetative development. Strikingly, exogenous GA₃ partially restored morphological defects while paradoxically intensifying amino acid mobilisation, indicating that GA‐dependent growth restoration occurs independently of metabolic normalisation. Together, these findings reveal that impaired amino acid biosynthesis imposes a fundamental constraint on plant growth and development, and that GA signalling can uncouple morphological recovery from metabolic homoeostasis. The differential expression of GA biosynthesis genes (*CPS*, *KS*, *GA3ox1*) coupled with upregulation of the starvation‐responsive gene *DIN6* in *dapat* mutant plants supports a model in which lysine availability is integrated into hormonal regulation of growth. Collectively, our findings suggest that primary metabolism functions as a master regulator integrating amino acid homoeostasis with hormonal signalling networks to coordinate growth and development.

## 2 Materials and Methods

### 2.1 Plant Material and Experimental Conditions

An *Arabidopsis thaliana* mutant line deficient in lysine biosynthesis, named *dapat* (Rate and Greenberg 2001), and its corresponding wild‐type (WT), both in the Columbia‐0 (Col‐0) background, were used in this study. Seeds were surface sterilised and imbibed for 4 days at 4°C in 1 mL of autoclaved deionized water in the dark. Following this, the seeds were transferred to 0.7% (*w/v*) agar plates containing half‐strength MS media (pH 5.7) (Murashige and Skoog 1962), supplemented with 1% sucrose, to germinate.

Seeds were germinated and grown under short‐day conditions (8 h light/16 h dark), at 20°C and 60% relative humidity. Afterwards, 12‐day‐old seedlings were transferred to individual 0.08 dm^3^ pots containing commercial substrate (Carolina Soil Standard, EC 0.7–8 Kg) and maintained under the same growth conditions for an additional 3 weeks.

### 2.2 *L,L*‐Diaminopimelate Aminotransferase Enzyme Activity Assay

*L,L*‐DAPAT enzyme activity was analyzed using the O‐amino benzaldehyde (OAB) assay adapted from Hudson et al. (2006). Frozen leaf material was ground into a fine powder using a ball mill. Proteins were extracted from 20 mg of leaf material by vortexing in 100 mM HEPES–*KO*H (pH 7.6). After centrifugation at 22,000 *g* for 15 min at 4°C, the supernatant was applied to an Amicon Ultra 30,000 MWCO filter unit (Millipore) for buffer exchange and concentration. The crude extract was further concentrated by centrifugation at 14,000 *g* for 30 min at 4°C and diluted twice with 450 μL of fresh 100 mM HEPES–KOH (pH 7.6). Protein concentration in the concentrated crude extract was determined using the Bradford protein assay kit (Bio‐Rad). DAPAT activity was measured by monitoring the increase in absorbance at 440 nm in a reaction mixture containing a total volume of 200 μL of 100 mM HEPES–KOH (pH 7.6), 0.5 mM *L,L*‐diaminopimelate (Sigma‐Aldrich, 89469), 2 mM 2‐oxoglutarate, 1,25 g ml^−1^ OAB (Sigma‐Aldrich, A9628) and the concentrated crude extract.

### 2.3 Gibberellin Application

Three‐week‐old plants were grown as described in Section 2.1, under an irradiance of 150 μmol photons m^−2^ s^−1^ and subjected to five applications of 20 μL of 25 mM gibberellic acid (GA₃; Sigma‐Aldrich G7645) and 0.05% Tween 20 over 14 days, at 72‐h interval (adapted from Paparelli et al. 2013). During the experiment, WT and *dapat* mutant plants were photographed at each application to evaluate visual phenotypes. At the end of the treatment, shoot samples were collected, immediately frozen in liquid nitrogen, and stored at ‐80°C until further analyses.

### 2.4 Biometric Parameters

Whole rosettes from 5‐week‐old plants were collected, and the following parameters were measured: rosette fresh mass (RFM), rosette dry mass (RDM), total rosette area (TRA), total leaf area (TLA), and specific leaf area (SLA). SLA was calculated using the following formula:

SLA = Leaf Disk Total Area (mm²)/Leaf Disk Dry Mass (mg)

The images obtained were processed using Image J software version 1.5.2 (Schneider et al. 2012).

### 2.5 Determination of Metabolite Levels

Leaves from the whole rosette of five individual plants were harvested 2 h before the end of the light (end of the day; ED) or dark (end of the night; EN) period. The harvest time was chosen based on the expression pattern of the *ent‐kaurene synthase* (*KS*) gene, which peaks in the afternoon of the diurnal cycle (Paparelli et al. 2013)

Methanolic extraction was conducted by rapidly grinding approximately 25 mg of fresh leaf tissue with liquid nitrogen, followed immediately by the addition of the extraction buffer. Chlorophyll contents (*a* and *b*) were determined as previously described (Porra et al. 1989). Starch (Fernie et al. 2001) and total protein levels (Bradford 1976) were measured in the insoluble fraction. In the soluble fraction, levels of organic acids (Nunes‐Nesi et al. 2007), glucose, fructose, and sucrose (Fernie et al. 2001), and total soluble amino acids (Gibon et al. 2004) were quantified.

### 2.6 Gene Expression Analysis

The expression of key genes involved in GA metabolism, amino acid metabolism, and alternative respiration pathways was analyzed using RT‐qPCR with the relative expression method. Total RNA was extracted from leaves of five individual plants with TRIzol reagent (Ambion, Life Technology). After purification, the RNA was quantified by NanoDrop ND‐2000 spectrophotometer (Thermo Scientific NanoDrop Technologies, Wilmington, Delaware, United States) and analyzed on a 1.5% (w/v) agarose gel. A total of 0.5 μg of RNA was treated with DNAse I (Invitrogen) to remove potential contamination with genomic DNA. The cDNA synthesis was performed from the four highest‐quality total RNA samples for each genotype/treatment, using 0.5 μg of total RNA using GoScript Reverse Transcriptase (A5003) with RNasin Ribonuclease Inhibitor and Oligo(dT) (A2791) following manufacturer protocol. For the analysis of gene expression, Ludwig qPCR SYBR‐green mix/ROX (ID:40) was used following the manufacturer protocol with the MicroAmpTM Optical 96‐well Reaction Plate (Applied Biosystems, Singapore, China) and MicroAmpTM Optical Adhesive Film (Applied Biosystems, Foster City, CA, USA). The relative expression levels were normalised using the reference gene *EF1a* (Wang et al. 2014). The primers used for RT‐qPCR were designed using the Primer3Plus web application (Untergasser et al. 2012) and are described in Supporting information S1: Table S1. The independent genotypes were represented by at least three plants, and the mean value of the expression was calculated by the relative‐expression method and represented as the normalised 2^‐(ΔCt)^ with its respective standard errors. The relative expression was then calculated as the average of three normalised 2^‐(ΔCt)^ values for each analyzed group.

### 2.7 Metabolite Profiling

Plant metabolites were extracted by adding 1.5 mL of ice‐cold extraction solvent (H₂O:MeOH:CHCl₃ at 1:2.5:1 v:v ratio) containing 50 μL of internal standards (5 mM Ribitol and DMPA) per 50 mL of buffer to each sample. Following vigorous vortexing (20 s), samples were incubated on an orbital shaker at 4°C for 10 min, then centrifuged at 20,000 *g* for 5 min at 4°C. The resulting supernatant (1.0 mL) was transferred to fresh microcentrifuge tubes and stored at −80°C until further analysis.

For GC‐MS analysis, five samples from individual plants per genotype/treatment were lyophilised (30 μL aliquots) using a speed‐vac concentrator prior to chemical derivatization (Gu et al. 2012; Shim et al. 2020). Mass spectral data were processed through sequential format conversion using ProteoWizard (mzXML output) and MetAlign (NetCDF conversion) with standard parameters. Automated deconvolution was performed using AMDIS (Automated Mass Spectral Deconvolution and Identification System, NIST), and putative metabolite identities were determined by comparing spectral matches against the NIST14 Mass Spectral Library with a similarity threshold of ≥ 70%. Compounds meeting this criterion were further validated against an in‐house reference standard collection. Peaks lacking confident identifications were designated based on their presumed biochemical class and chromatographic retention index.

Chromatographic peak areas were determined using MassHunter Quantitative software (v b08.00, Agilent Technologies). All metabolite abundances were normalised relative to sample fresh mass and the Ribitol internal standard (Sigma‐Aldrich) to account for instrumental and extraction variability.

### 2.8 Experimental Design and Statistical Analyses

The experimental design was a completely randomised 2 × 2 factorial design, consisting of two genotypes (WT and *dapat* mutant plants) and two treatments (control and GA_3_ application). Statistical analyses were conducted using two‐way ANOVA, followed by Tukey's HSD test at 5% significance level for mean comparison. Additionally, the relative gene expression analysis was performed using Student's *t*‐test, with a significant level of 5% (*α* = 0.05), using WT‐GA as control. Statistical tests were conducted separately for the end of day (ED) and end of night (EN) time points.

## 3 Results

### 3.1 GA‐Mediated Compensation of Growth Defects in *Dapat* Mutants Occurs Independently of DAPAT Enzyme Function

**Figure 1:**
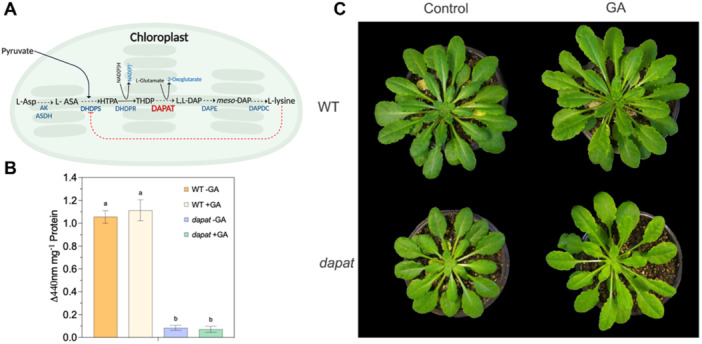
Characterisation of the *dapat* mutant: Lysine biosynthesis deficiency and phenotypic response to Gibberellin. (A) Schematic representation of Lysine biosynthesis in the chloroplast, highlighting the role of *L,L*‐diaminopimelate aminotransferase (DAPAT) enzyme; (B) DAPAT enzyme activity assay performed under control conditions and following GA_3_ treatment, (C) Representative phenotype of 36‐day‐old plants grown under control conditions and after GA_3_ treatment. Letters indicate significantly different values as determined by Tukey's test at the 5% significance level, with comparisons made within each column. Values represent the mean ± standard error of six independent samples.

To elucidate the phenotypic consequences of impaired lysine biosynthesis, we employed the *A. thaliana dapat* mutant, a known EMS‐induced line carrying a G → A point mutation within the DAPAT locus (Song et al. 2004). *DAPAT* encodes one of the enzymes involved in lysine biosynthesis, the *L,L*‐DAPAT (Figure 1A). This mutation results in a missense substitution, where the amino acid proline is replaced by a serine residue at position 398 (exon 10), leading to less ~90% enzyme activity (Figure 1B), and impaired plant growth (Figure 1C).

**Figure 2:**
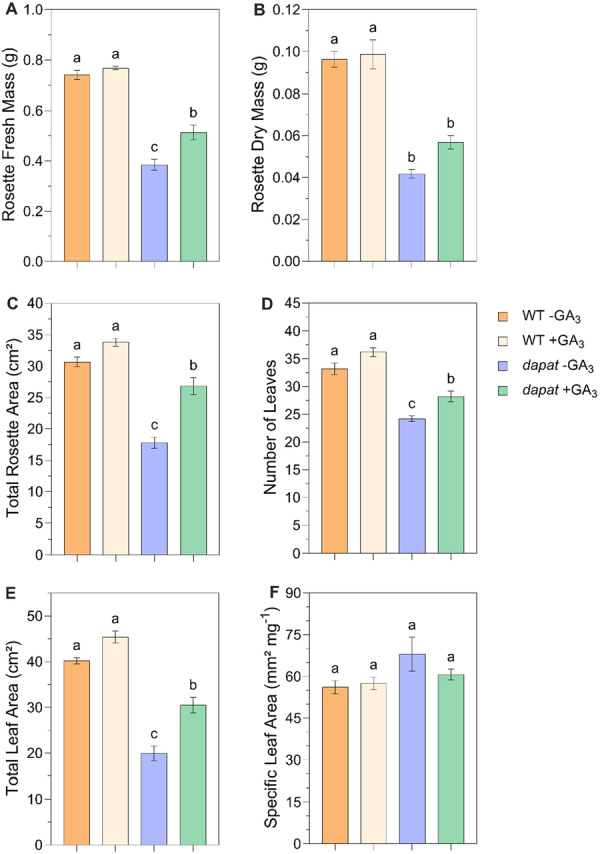
Gibberellin treatment rescues the rosette growth defects of lysine‐deficient *dapat* mutant plants. Biometric measurements of wild‐type (WT) and *dapat* under control conditions (control) and following GA_3_ treatment (+ GA). Parameters measured include rosette fresh mass (A), rosette dry mass (B), total rosette area (C), number of leaves (D), total leaf area (E), and specific leaf area (F). ANOVA followed by Tukey's HSD test at the 5% significance levels was used for mean comparison. Statistical tests were conducted separately for measurements taken at the end of the day (ED) and end of the night (EN). Values represent the mean ± standard error of five independent samples. [Color figure can be viewed at wileyonlinelibrary.com]

Exogenous GA_3_ application seemingly alleviates the growth impairments in the lysine‐deficient mutant plants without impacting DAPAT enzyme activity (Figure 1B). GA application alone led to an increase in *dapat* mutant rosette growth, nearly matching the growth of untreated WT plants (Figure 1C). To further investigate the impact of the DAPAT mutation on plant growth, we compared the development of WT and *dapat* plants. Consistent with previous findings (Cavalcanti et al. 2018; Song et al. 2004), *dapat* exhibited a reduced rosette diameter (~ 42% ‐ Figure 1C; Figure 2), accompanied by a notable decrease in both fresh and dry mass accumulation (48% and 57% respectively ‐ Figure 2); as well, as a reduction in total leaf area (~ 50%) compared to WT plants (Figure 2).

GA treatment enhanced fresh mass accumulation (Figure 2A), rosette growth (Figure 2C), number of leaves (Figure 2D, see Supporting Figure S1), leaf area (Figure 2E), and specific leaf area (SLA, Figure 2F) in *dapat* plants, although it did not significantly increase dry mass (Figure 2B). While GA application positively impacted almost all measured parameters in mutant plants, it had minimal impact on WT, suggesting an enhanced response of *dapat* to GA treatment (Figure 1C; Figure 2A–F; see Supporting Information S1: Figure S2).

### 3.2 Gibberellin Influences Sugar Starvation and Metabolic Remodelling in the *Dapat* Mutant

**Figure 3:**
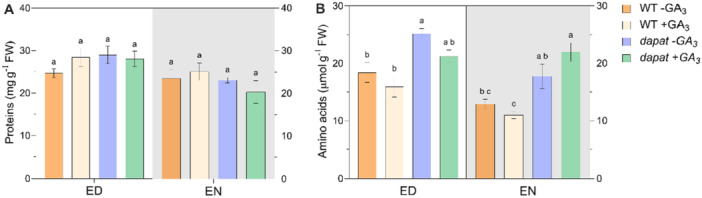
Exogenous Gibberellin exacerbates amino acid accumulation in *dapat* mutant plants without altering protein content. Variation in nitrogen compounds levels in *Arabidopsis thaliana* under control conditions (control) or following GA_3_ treatment (+ GA). Total proteins (A), and total amino acids (B) were measured at the end of the day (ED, white background) and the end of the night (EN, grey background). Statistical analyses were conducted using two‐way ANOVA, followed by Tukey's HSD test at 5% significance levels for mean comparison. Statistical tests were performed separately for ED and EN time points. Values represent the mean ± standard error of five independent samples. [Color figure can be viewed at wileyonlinelibrary.com]

To further understand the effect of exogenous GA on primary metabolism in lysine deficient plants, we measured metabolites at the end of the day (ED) and the end of the night (EN). Specifically, we assessed pigments, total protein and amino acid contents, reducing sugars, sucrose, starch, and organic acids levels. Pigment quantification (chlorophylls a and b, carotenoids) revealed that GA treatment reduced chlorophyll a and b levels in WT plants at ED, with no changes in either genotype at EN (Supporting Information S1: Figure S3A and S3B). In contrast, *dapat* mutant showed lower carotenoid levels under control conditions at ED, while GA presence decreased carotenoids in WT at the same timepoint but had no effect in *dapat* (Supporting Information S1: Figure S3C). *dapat* mutant plants accumulated elevated free amino acids at the ED compared to WT control (Figure 3B), while total protein remained stable across genotypes (Figure 3A). Notably, exogenous GA further enhanced nighttime amino acid accumulation in *dapat* plants (Figure 3B), suggesting that GA supplementation exacerbates nitrogen accumulation despite growth stimulation.

**Figure 4:**
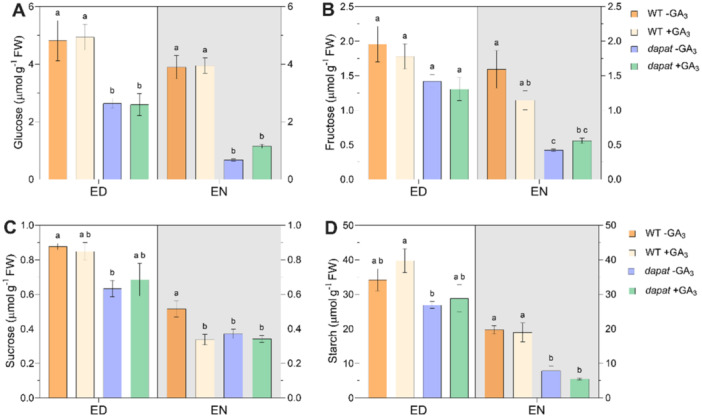
*Dapat* mutant plants display a constitutive carbon starvation profile that is unresponsive to Gibberellin supplementation. Variation in carbohydrate contents in *Arabidopsis thaliana* under control conditions (control) or following GA_3_ treatment (+ GA). Glucose (A), Fructose (B), Sucrose (C), and Starch (D) were measured at the end of the day (ED, white background) and the end of the night (EN, grey background). Statistical analyses were performed using two‐way ANOVA, followed by Tukey's HSD test at 5% significance level for mean comparison. Statistical tests were performed separately for ED and EN time points. Values represent the mean ± standard error of five independent samples. [Color figure can be viewed at wileyonlinelibrary.com]

**Figure 5:**
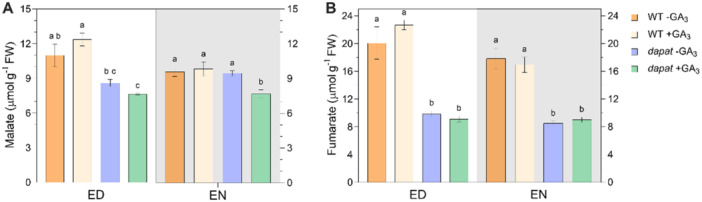
Depletion of TCA cycle intermediates reveals impaired carbon storage in *dapat* mutant unresponsive to Gibberellin. Variation in organic acid content in *Arabidopsis thaliana* under control conditions (control) or following GA_3_ treatment (+ GA). Malate (A) and Fumarate (B) were measured at the end of the day (ED, white background) and the end of the night (EN, grey background). Statistical analyses were performed using two‐way ANOVA, followed by Tukey's HSD test at 5% significance level for mean comparison. Statistical tests were performed separately for ED and EN time points. Values represent the mean ± standard error of five independent samples. [Color figure can be viewed at wileyonlinelibrary.com]

Glucose and fructose levels were consistently reduced in *dapat* plants at both ED and EN compared to WT (Figure 4A,B). Sucrose depletion in mutant plants persisted across both timepoints and remained insensitive to GA treatment, contrasting with WT plants, which exhibited GA‐dependent sucrose reduction at EN (Figure 4C). Similarly, starch accumulation followed the same pattern, with *dapat* exhibiting lower levels at ED and EN that remained unresponsive to GA application (Figure 4D). Malate levels were reduced in *dapat* plants at the ED relative to WT, whereas no significant differences were observed between genotypes at the EN under control conditions (Figure 5A). Notably, GA_3_ treatment induced further malate depletion in *dapat* at the EN, suggesting that supplementation exacerbates malate deficiency in the mutant (Figure 5A). In contrast, fumarate concentrations were significantly diminished in *dapat* at both ED and EN points and remained unaffected by GA (Figure 5B).

### 3.3 Persistent Metabolic Imbalance in *Dapat* Mutant: Uncoupling of GA‐Mediated Growth From Carbon‐Nitrogen Metabolism

**Figure 6:**
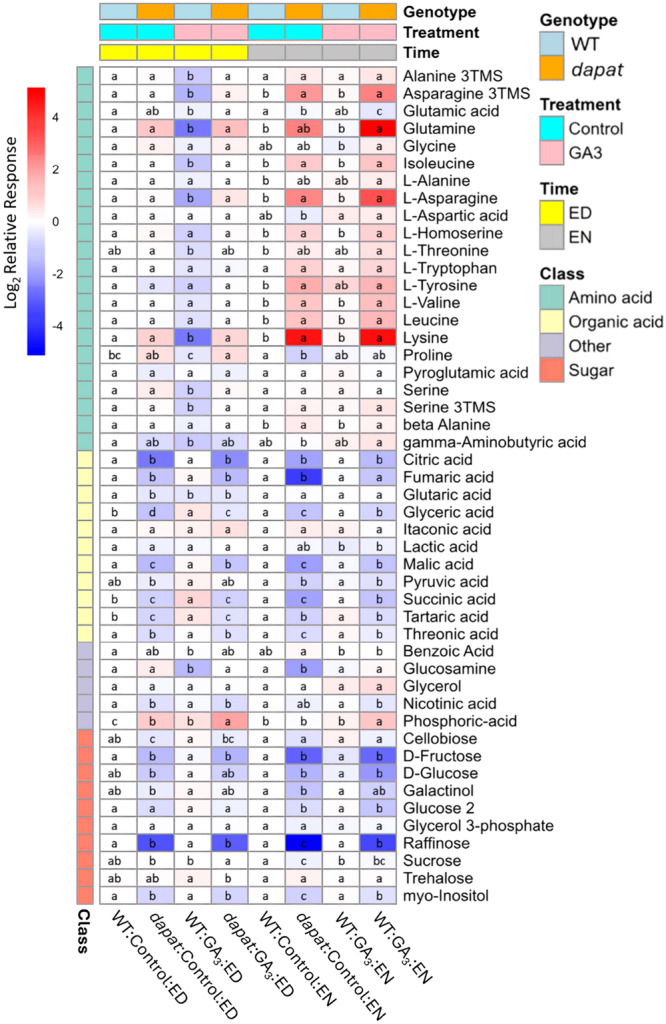
Global metabolomic profiling reveals a shift from carbon storage to amino acid accumulation in *dapat* mutant plants. 5‐week‐old *Arabidopsis thaliana* leaves across two genotypes (WT and *dapat* mutant) and two treatments: Control and GA_3_ treatment, harvested 2 h after the end of the day (ED) and 2 h after the end of the night (EN). Each cell represents the log₂ fold change in metabolite levels normalised to WT:Control treatment. Compact letter displays within each cell indicate statistically significant differences between groups based on two‐way ANOVA followed by Tukey's HSD test at a 5% significance level. Colours represent the log₂ fold change: red indicates enrichment, blue indicates depletion, and white represents no change. Genotype colours are light blue (WT) and orange (*dapat*). Treatment colours are represented in the top bar as cyan for Control and light red for GA_3_. Metabolite classes are indicated in the right bar: green for amino acids, pale yellow for organic acids, red for sugars, and light purple for other metabolites. Values represent the mean ± standard error of five independent samples. [Color figure can be viewed at wileyonlinelibrary.com]

To systematically characterise the metabolic alterations triggered by the DAPAT mutation and assess the response to exogenous GA, we performed comprehensive metabolomic profiling of leaf samples collected at both the ED and EN timepoints. Metabolomic profiling revealed distinct metabolic signatures between WT and *dapat* plants. Under control conditions at the ED, amino acid content did not differ significantly between genotypes, with lysine levels remaining unchanged despite the lysine biosynthesis impairment in the mutant (Figure 1A). By contrast, the EN samples showed pronounced amino acid accumulation in *dapat* leaves, including elevated asparagine, glutamine, lysine, branched‐chain amino acids (BCAA: leucine, isoleucine, valine), homoserine, threonine, tyrosine, and tryptophan, alongside reduced proline and glutamic acid (Figure 6), consistent with the total amino acid accumulation observed (Figure 3).

GA application at the ED induced global amino acid depletion in WT plants relative to both untreated controls and GA‐treated *dapat* mutant, with marked reductions in lysine, glutamine, asparagine, serine, and isoleucine (Figure 6). By contrast, GA application failed to restore amino acid homoeostasis in *dapat*, with mutant plants maintaining markedly elevated amino acid levels at the EN, including intensified accumulation of lysine, glutamine, and asparagine alongside suppressed glutamic acid content (Figure 6). WT plants showed modest accumulation of aspartic acid and tyrosine at the EN following GA supplementation, further highlighting the genotype‐dependent metabolic response to GA.

Concurrent analysis of organic acids and sugars revealed at the ED under control conditions a global reduction in organic acids in *dapat* plants compared to WT, including citric, fumaric, glyceric, malic, pyruvic, and succinic acids, with concomitant accumulation of nicotinic and phosphoric acids (Figure 6). Glucose and fructose concentrations were reduced, while raffinose showed pronounced depletion in the mutant (Figure 6). Similar patterns persisted at the EN, with maintained reductions in organic acids and sugars, particularly raffinose. GA_3_ treatment elicited subtle increases in select organic acids ‐ glyceric, pyruvic, succinic, tartaric, and phosphoric acids ‐ in WT plants, whereas sugar profiles remained comparable to control conditions (Figure 6). The *dapat* mutant sustained metabolite depletion patterns equivalent to untreated plants, with organic acid and sugar deficiencies, particularly raffinose (Figure 6). During the night, *dapat* exhibited sustained decrease of organic acids, glucose, fructose, and raffinose despite GA‐mediated growth restoration (Figure 6), indicating dissociation between growth rescue and restoration of carbon‐nitrogen metabolic balance.

### 3.4 Linking GA and Lysine Biosynthesis to Growth Impairments

**Figure 7:**
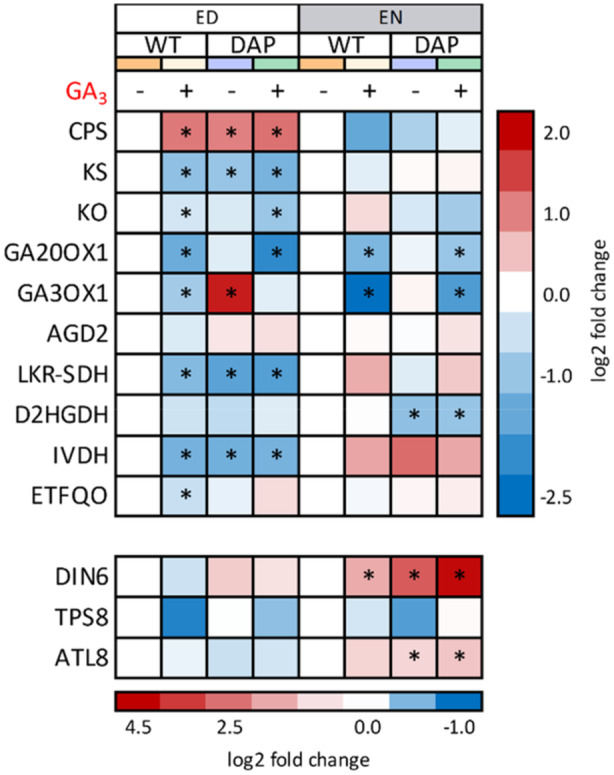
The DAPAT mutation causes significant differential expression of genes involved in Gibberellin biosynthesis at the ED. Changes in the expression levels of genes encoding enzymes involved in GA biosynthesis and amino acids metabolism in *Arabidopsis thaliana* under control conditions (‐GA_3_) or following GA_3_ treatment (+ GA_3_). RT‐qPCR analysis of transcript levels of *CPS* gene, *KS*, *KO*, *GA20ox1*, *GA3ox1*, *AGD2* (*DAPAT* gene), *LKR/SDH, D2HGDH, IVDH, ETFQO*, *DIN6*, *TPS8* and *ATL8*. Expression levels are shown as Log_2_ Fold Change at the end of the day (ED) and end of the night (EN) compared to WT‐GA_3_. Asterisks (*) indicate significant differences by Student's *t*‐test compared to WT‐GA_3_ within each time group, at 5% of significance levels. Data represents the mean of three independent samples ( ± SEM) and the reference group for the ΔΔCt method used is wild‐type for each time point analyzed. Additionally, RT‐qPCR relative expression plots are available in Supporting Information S1: Figures S4 and S5. [Color figure can be viewed at wileyonlinelibrary.com]

The ability to partially rescue the dwarf phenotype of the mutant plants suggests an impact on GA metabolism. To investigate the contribution of GA biosynthesis to the phenotype of the lysine‐deficient *dapat* mutant, we evaluated representative genes involved in GA biosynthesis, lysine biosynthesis/degradation, and alternative respiratory pathways at the ED and EN (Figure 7; Supporting Information S1: Figures S4 and S5).

A 2.0‐fold reduction in *KS* transcript levels was observed in *dapat* control plants compared to WT at the ED (Figure 7). Similarly, most transcripts of the GA‐related genes analyzed (e.g., *KS*, *KO*, and *GA20ox1*) were 2.0 to 4.0‐fold less abundant in GA‐treated plants.

Transcripts of genes encoding key enzymes at the start (*CPS*) and at the end (*GA3ox1*) of the GA biosynthesis pathway were elevated in the *dapat* mutant under control conditions. Given that *CPS* catalysers the initial step in GA biosynthesis, its expression likely represents a critical regulatory point activating this pathway. Upon treatment at the EN, *GA3ox1* expression was significantly downregulated in both WT (~ 5.6‐fold) and *dapat* (~3.3‐fold) plants. Similarly, *GA20ox1* was downregulated by approximately 2.4‐ and 2.0‐fold in WT and *dapat*, respectively (Figure 7).

### 3.5 Lysine Catabolism Gene Expression Responds to GA Application

Considering the observed alterations in carbohydrate and nitrogen metabolism, we quantified transcript levels of key catabolic genes *LKR/SDH*, *IVDH*, *D2HGDH*, and *ETFQO*. At the ED, transcripts of *LKR/SDH* and *IVDH* were reduced by at least 2.0‐fold in all plants relative to the WT control, reflecting the impact of GA_3_ treatment (Figure 7). In contrast, expression of *D2HGDH* and *ETFQO* remained statistically unchanged irrespective of genotype or treatment condition (Figure 7). During the EN, *LKR/SDH* was significantly upregulated (~ 1.9‐fold) in GA‐treated WT plants, whereas *D2HGDH*, *IVDH*, and *ETFQO* exhibited modest downregulation trends across both control and GA‐treated groups (Figure 7).

### 3.6 Sugar Starvation and Gene Expression Shifts in *Dapat* Mutant Leaves

Impaired growth and significantly reduced carbohydrate levels in *dapat* under control and GA‐treated conditions prompted measurement of starvation‐related gene expression. *DIN6 (*Nozawa et al. 1999 *)*, *ATL8* (Luo et al. 2019; Graf et al. 2010), and *TPS8* (Kolbe et al. 2005) transcript levels were assessed.

TPS8 (Trehalose‐6‐Phosphate Synthase 8), a class II member of the *TPS* gene family that functions as a carbohydrate‐status sensor through trehalose‐6‐phosphate (T6P) signalling (Lunn et al. 2006; Fichtner and Lunn 2021), was downregulated in *dapat* mutant plants at the EN (Figure 7). However, the *TPS8* suppression remained unresponsive to GA₃ treatment, indicating that GA signalling does not regulate *TPS8* expression in lysine‐deficient plants.

**Figure 8:**
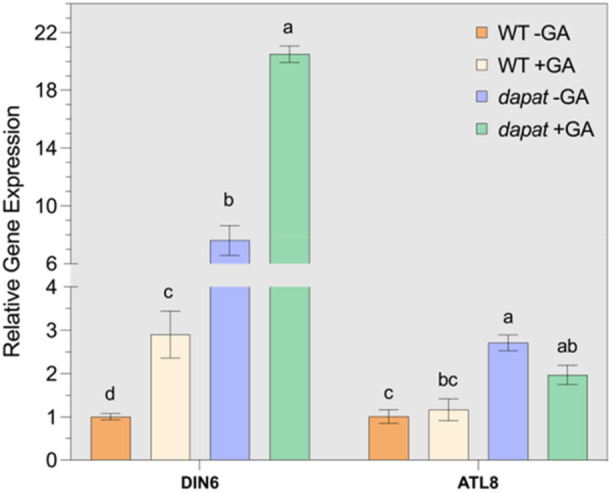
Gibberellin treatment exacerbates the carbon starvation response in *dapat* mutant as revealed by *DIN6* strong upregulation at the EN. Relative expressions of *DIN6* and *ATL8* as markers of the sugar starvation process. Comparisons of the expression of the starvation reporter genes *DIN6* (A) and *ATL8* (B) in *Arabidopsis thaliana* under control conditions (control) or following GA_3_ treatment (+ GA). Statistical analyses were conducted using ANOVA, followed by Tukey's HSD test at 5% significance level for mean comparison. Statistical tests were performed separately for the end of the day (ED) and end of the night (EN) time points. Data represents the mean of three replicates ( ± SEM) and the reference group for the ΔΔCt method used is wild‐type for each time point analyzed. [Color figure can be viewed at wileyonlinelibrary.com]

Notably, *DIN6* expression showed no differential regulation at the ED across genotypes and treatments; however, this starvation‐responsive gene exhibited pronounced upregulation in *dapat* mutant leaves specifically during the EN under optimal growth conditions, providing compelling evidence of marked sugar starvation (Figures 7 and 8). GA supplementation further intensified *DIN6* upregulation in *dapat* plants, revealing that exogenous GA exacerbates the starvation‐like metabolic state, likely through stimulated growth and increased energy demands. WT plants, by contrast, showed a slight *DIN6* upregulation following GA₃ treatment (Figure 8).

These findings are reinforced by *ATL8* expression patterns: *dapat* mutant leaves exhibited elevated *ATL8* transcription during the night, providing additional evidence of constitutive sugar starvation (Figures 7 and 8) as a consequence of DAPAT mutation (Figure 1B).

### 3.7 Multivariate Analysis Reveals Decoupling of Growth Rescue and Metabolic Homoeostasis in *Dapat* Mutant Plants

While individual physiological, metabolic, and transcriptional analyses revealed pronounced genotype‐ and treatment‐dependent effects, these datasets do not fully capture how growth, metabolism, and gene expression are coordinated at a systems level. To integrate these multidimensional responses and assess whether GA treatment alters the metabolic identity imposed by the DAPAT mutation, we conducted a principal component analysis (PCA) combining metabolomic, and transcriptomic variables across genotypes, treatments, and time points (Supporting Information S1: Figures S6 and S7). This multivariate approach revealed distinct metabolic signatures associated with both the DAPAT mutation and GA responsiveness throughout the diel cycle. The first four principal components collectively explained 73.35% of the total variance, with PC1 and PC2 accounting for 36.27% and 22.12%, respectively (Supporting Information S1: Figures S6).

PC1 primarily separated samples along a metabolic‐energetic axis, with high positive loadings for sugars (glucose, fructose, sucrose), organic acids (fumarate, malate), and key transcripts associated with carbon partitioning and catabolism (e.g. *LKR/SDH*, *IVDH, D2HGDH*). This dimension distinguishes samples with high carbon availability and active respiratory metabolism from those with reduced sugar availability and diminished catabolic capacity, reflecting the fundamental metabolic constraint imposed by lysine biosynthesis deficiency in *dapat*. In contrast, PC2 captured the effects of GA_3_ treatment and diel variation. The differential separation of the ED and EN points along this axis underscores circadian‐regulated metabolic remodelling, particularly highlighting enhanced nighttime mobilisation of stored carbohydrates and the diurnal accumulation of biosynthetic precursors.

Key metabolite loadings driving this multivariate structure included carbohydrates (glucose, fructose, sucrose, starch), organic acids (fumarate, malate, citrate), amino acids, and pigments (Supporting Information S1: Figure S7), underscoring the constitutive stress‐like metabolic state of *dapat* plants characterised by impaired carbon storage and altered energy partitioning.

Hierarchical clustering of all biometric, metabolic, and transcriptomic variables (Supporting Information S1: Figures S8 and S9) confirms this scenario, revealing profound and sustained metabolic dysregulation in the *dapat* mutant alongside the selective efficacy of GA₃ treatment. Notably, samples clustered primarily by genotype along the vertical axis, with WT and *dapat* plants forming distinct clades that remained separate despite GA₃ application. This indicates that the DAPAT mutation establishes a dominant metabolic and transcriptional state that cannot be fully overridden by GA‐mediate growth restoration.

## 4 Discussion

Beyond its canonical role as substrate for protein biosynthesis, lysine acts as a central metabolic hub, linking amino acid catabolism with mitochondrial energy production and stress responses (Araújo et al. 2010, 2011; Galili 2016; Hildebrandt 2018; Yang et al. 2020; Neves et al. 2024; Singh et al. 2026). The embryonic lethality of *dapat* null mutant reveals that lysine biosynthesis is essential for plant development, indicating that this pathway cannot be functionally compensated (Song et al. 2004). *dapat* knockdown mutant plants, characterised by significantly reduced *L,L*‐DAPAT enzymatic activity (Figure 1B), exhibits stunted growth and extensive rewiring of metabolic networks (Cavalcanti et al. 2018; Neves et al. 2024), including constitutive salicylic acid accumulation and enhanced *Pseudomonas syringae* resistance (Rate and Greenberg 2001; Song et al. 2004). This pattern is consistent with a growth‐defence metabolic trade‐off, wherein developmental progression is subordinated to stress defence, maintaining a constitutive “stressed” phenotype even under non‐stressful conditions.

Based on earlier finding (Cavalcanti et al. 2018), we hypothesised that exogenous GA application (GA_3_) might alleviate growth limitations in *dapat* plants, providing an opportunity to investigate how GA‐dependent growth promotion interacts with to metabolic constraints imposed by lysine biosynthesis. Indeed, GA_3_ partially rescued shoot elongation and leaf expansion in *dapat* plants (Figure 1C), though root biomass remained unaffected (Figure 2B). This tissue‐specific morphological recovery supports our hypothesis that GA‐mediated growth promotion alleviates developmental constraints imposed by lysine deficiency. However, the partial rescue of growth by GA_3_ treatment occurs within the context of persistent metabolic imbalance in *dapat* mutant plants (Figure 6; Cavalcanti et al. 2018).

Metabolomic analysis positioned amino acid metabolism at the core of the *dapat* phenotype, revealing its integrative role in linking lysine biosynthesis deficiency, carbon limitation, and GA‑stimulated growth. Although amino acid levels were indistinguishable between genotypes at the end of the day (ED), *dapat* plants exhibited a distinct, constitutive amino acid accumulation during the night (EN) (Figure 3B). This profile included elevated asparagine, glutamine, and branched‐chain amino acids (BCAAs), showing elevated lysine accumulation alongside reduced *de novo* synthesis capacity (Figure 6). These metabolic signatures closely mirror sugar starvation stress (Ishizaki et al. 2005, 2006; Pires et al. 2016; Barros et al. 2017; Hirota et al. 2018), wherein amino acid catabolism sustains mitochondrial respiratory demand under carbon‐limited conditions.

Consistently, *dapat* mutants displayed persistent depletion of soluble sugars (glucose, fructose, sucrose, and raffinose) and reduced starch accumulation throughout the diurnal cycle, with no recovery upon GA₃ supplementation (Figure 4). In contrast, WT plants adjust sucrose levels under GA treatment at EN, suggesting flexible behaviour under GA regime (Figure 6). In parallel, the robust upregulation of canonical sugar‐starvation markers *DIN6* (Nozawa et al. 1999; Fujiki et al. 2001; Gonzali et al. 2006; Contento et al. 2010) and *ATL8* (Graf et al. 2010; Luo et al. 2019) confirmed a constitutive nighttime starvation in *dapat* plants, further exacerbated by GA₃ treatment (Figure 8).

These findings parallel previous observations in starch‐deficient mutants where impaired nighttime carbohydrate supply, coupled with GA biosynthetic defects, led to growth suppression and altered carbon allocation (Paparelli et al. 2013). In *dapat*, however, GA supplementation fails to reestablish C/N balance; instead, it forces a compensatory accumulation of amino acids likely as a compensatory response to increased energy demands for growth. This underscores a breakdown in the coordination between hormonal signalling and carbon availability described by Paparelli et al. (2013).

This shift toward amino acid reliance is further evidenced by widespread depletion of organic acids. *dapat* plants exhibit reduced levels of several TCA cyle intermediates at ED, with this pattern persisting into the night (Figure 6). Fumarate, a critical respiratory and carbon storage intermediate during carbon deprivation (Chia et al. 2000; Fahnenstich 2008; Gibon et al. 2009; Zell et al. 2010; Araujo et al. 2012), is consistently diminished at both timepoints and remains insensitive to GA (Figure 5B), while malate becomes further depleted in *dapat* at EN following GA_3_ treatment (Figure 5A). Such patterns are consistent with the reallocation of respiratory substrates from TCA intermediates towards amino acid metabolism, thereby sustaining mitochondrial ATP production when carbohydrate supply is limited (Pracharoenwattana et al. 2010).

Building on prior findings, transcript levels of alternative respiration pathway genes revealed constitutive repression of *IVDH* and *LKR/SDH* in *dapat* plants at ED, persisting regardless of GA₃ treatment (Figure 7; Supporting Figures S4 and S5). This indicates that exogenous GA₃ fails to normalise BCAA and lysine catabolism at this time point (Araújo et al. 2010, 2011; Hildebrandt et al. 2015; Heinemann and Hildebrandt 2021). At EN, *D2HGDH* remains consistently downregulated in mutants under both conditions, pointing stable disruption of 2‐hydroxyglutarate (HG) metabolism (Engqvist et al. 2014), while *IVDH* and *LKR/SDH* show a tendency for upregulation in the mutant under GA_3_ supplementation, consistent with enhanced nighttime catabolic activation (Figure 7). These patterns align with the alternative respiratory pathway described by Araújo et al. (2010), wherein amino‑acid degradation feeds the tricarboxylic acid cycle under carbon limitation. This GA‐exacerbated starvation, driven by enhanced mobilisation of amino acids as alternative respiratory substrates, suggest an accelerated proteolytic and oxidative catabolism to meet growth‐related energy demands (Ishizaki et al. 2005; Araújo et al. 2011; Hildebrandt et al. 2015). Ultimately, our findings highlight the role of lysine biosynthesis as a key metabolic checkpoint, coordinating amino acid homoeostasis, carbon metabolism, and hormonal regulation to support balanced plant development.

*Nighttime sugar starvation disrupts GA biosynthesis gene dynamics despite GA‐responsive growth*

The altered transcriptional profile of GA biosynthesis genes in *dapat* plants provides mechanistic insights into the interplay between energy status and hormone metabolism. In WT plants, *KS* expression peaks at ED, consistent with its regulation by diurnal carbohydrate dynamics. This peak is markedly attenuated in *dapat* and remains low even after GA₃ supplementation (Figure 7), consistent with *KS* repression under carbon‐limited conditions (Paparelli et al. 2013). This suppression suggests that energy deficiency overrides GA feedback regulation at this critical biosynthetic step. In contrast, *CPS* and *GA3ox1* are upregulated in *dapat* mutant plants under control conditions, revealing node‐specific responses within the GA biosynthetic pathway to the perturbed metabolic state. The uncoupling of *CPS* from *KS* suggests that upstream steps are less tightly constrained by sugar‑dependent feedback (Fleet et al. 2003), whereas elevated *GA3ox1* expression likely represents a compensatory response to presumed bioactive GA insufficiency (Rieu et al. 2008; Martin et al. 1996).

**Figure 9:**
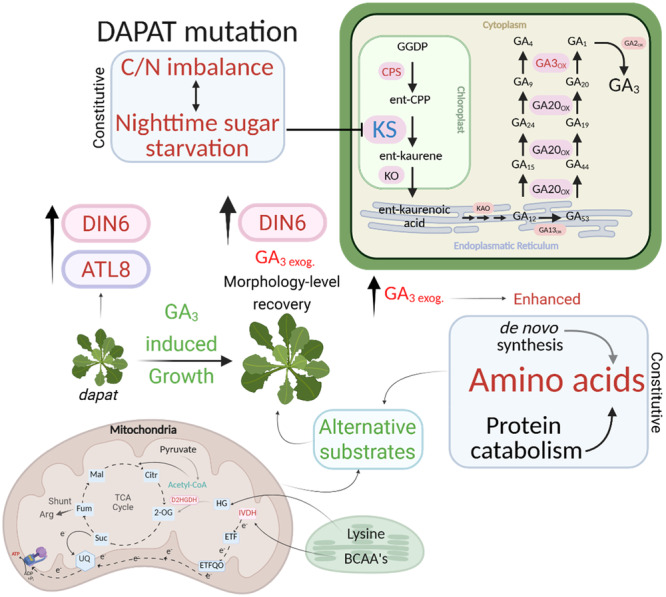
Model illustrating the interplay between C/N imbalance, repressed GA biosynthesis, and the metabolic cost of forced growth in *dapat* mutant plants. Schematic overview of key metabolic pathways affected by the DAPAT mutation, with emphasis on sugar starvation and amino acid mobilisation. Gibberellin (GA) biosynthesis pathway is highlighted within the green‐outlined box. Enzymes with expression profiles analyzed at the end of the day (ED) and end of the night (EN) are shown in pink ellipses with blue text. The *dapat* mutant exhibits a constitutively altered metabolic state characterised by persistent depletion of soluble sugars (glucose, fructose, sucrose), starch, and tricarboxylic acid (TCA) cycle intermediates (e.g., malate, fumarate), at both timepoints. This reflects impaired carbon assimilation, restricted storage capacity, and enhanced respiratory demand. Concomitantly, *dapat* mutant plants accumulated high levels of amino acid indicating a shift toward amino acid catabolism to support mitochondrial energy production and growth. Dysregulation of GA biosynthetic gene expression (*CPS*, *KS*, *GA3ox1*) reveals a functional connection between lysine metabolism and GA signalling. Although GA supplementation partially rescues morphological defects, it fails to normalise metabolic homoeostasis, instead exacerbating amino acid mobilisation. These findings demonstrate that *DAPAT* activity is essential not only for lysine biosynthesis but also for maintaining C/N balance and coordinating energy metabolism with hormonal growth cues. [Color figure can be viewed at wileyonlinelibrary.com]

The upregulation of *GA3ox1* in *dapat* plants constitutes a failed compensatory mechanism triggered by 2‐oxoglutarate (2‐OG) substrate limitation. GA biosynthetic enzymes are 2‐OG‐dependent dioxygenases, and their catalytic capacity is constrained by substrate availability (Araújo et al. 2014). In fact, increased *GA3ox1* expression cannot overcome 2‐OG depletion caused by enhanced amino acid catabolism and reduced *KS* transcript levels linked to nighttime carbohydrate limitation (Paparelli et al. 2013). Together, these findings support a model in which the DAPAT mutation imposes a chronic sugar‑starvation that repress *KS*, thereby limiting GA biosynthesis, despite the transcriptional compensation via *CPS* and *GA3ox1* observed in *dapat*. Consequently, growth‑promoting signals are perceived and partially executed via exogenous GA that partially restores growth, yet this recovery is accompanied by further induction of amino acid catabolism, including enhanced *DIN6* expression and depletion of TCA intermediates, suggesting increased respiratory demand under energetic stress. This metabolic phenotype is consistent with the alternative respiratory pathway described previously (Araújo et al. 2010), wherein amino‑acid degradation feeds the TCA cycle under carbon limitation. Together, our findings support a model in which lysine biosynthesis deficiency in *dapat* mutant plants triggers GA‑modulated reprogramming of amino‑acid catabolism to sustain mitochondrial energy metabolism, thereby exacerbating the C/N imbalance (Figure 9), uncoupling morphological growth from metabolic homoeostasis that characterises lysine‑deficient plants (Cavalcanti et al. 2018; Neves et al. 2024).

## 5 Conclusions

This study reveals lysine metabolism as a critical metabolic checkpoint coordinating energy status, stress responses, and hormone‐dependent growth regulation in plants. We demonstrate that the enzyme *L,L*‐DAPAT, a key lysine biosynthetic enzyme, play a role beyond lysine production, acting as a metabolic integrator that links C/N balance to growth‐promoting GA signalling. The *dapat* mutant, characterised by impaired lysine synthesis, exhibits constitutive markers of energy deprivation, including depleted carbohydrate reserves, elevated amino acid pools, and transcriptional signatures indicative of sugar starvation, even under optimal growth conditions. These findings suggest that lysine biosynthesis deficiency triggers a persistent stress‐like metabolic state, redirecting resources from growth towards stress tolerance.

Importantly, we uncover a previously unrecognised link between lysine biosynthesis and GA metabolism: altered diurnal expression of GA biosynthesis genes (*CPS, KS, GA3ox*) in *dapat* mutant plants suggests that lysine availability influences hormone biosynthetic gene dynamics. Notably, exogenous GA_3_ supplementation rescues the *dapat* growth‐inhibition phenotype, positioning GA as a downstream effector linking lysine metabolism to development and suggesting that amino acid availability regulates developmental plasticity via hormone biosynthesis.

The persistence of metabolic imbalance despite GA_3_‐induced morphological recovery underscores the complexity of lysine metabolism role: it functions not only as a metabolic substrate but likely also as a systemic regulator influencing source‐sink dynamics. Through multivariate analyses, we show that amino acid accumulation reconfigures metabolic networks in favour of respiratory compensation, reinforcing the prioritisation of survival over growth. Together, this work establishes lysine metabolism as a regulatory hub at the interface of primary metabolism and hormone signalling. It also open new avenues for future research, particularly exploiting improving nutrient‐use efficiency and stress resilience by targeting amino acid–hormone, particularly under fluctuating environmental and nutritional conditions.

## Conflicts of Interest

The authors declare no conflicts of interest.

## Supporting information

Supporting File

## Data Availability

The data that support the findings of this study are available from the corresponding author upon reasonable request.
