# Supplementary material for: Lysine Biosynthesis Defines a Metabolic Checkpoint for Gibberellin‐Mediated Growth in Arabidopsis thaliana
Source: Plant Cell Environ. 2026 Apr 16;49(8):5343–57. doi: 10.1111/pce.70525 (PMC13353673; doi:10.1111/pce.70525)
Supplement: Supplementary file 1 — Supporting File [file PCE-49-5343-s001.docx]

**Supporting Information**

**Supplementary Figure S1**. GA regime positively impacts the number of leaves in the *dapat* mutant plants.

**Supplementary Figure S2.** Gibberellin partially restores biomass allocation in the lysine-deficient *dapat* mutant plants.

**Supplementary Figure S3.** GA treatment negatively impacts pigment content in both wild-type (WT) and *dapat* mutant plants.

**Supplementary Figure S4.** Relative expression levels of genes encoding enzymes involved in GA and amino acids metabolism at the end of the day.

**Supplementary Figure S5**. Relative expression levels of genes encoding enzymes involved in GA and amino acids metabolism at the end of the night.

**Supplementary Figure S6**. Principal component analysis (PCA) of metabolite levels in *Arabidopsis thaliana* wild-type (WT) and *dapat* at the end of the day (ED).

**Supplementary Figure S7**. Principal component analysis (PCA) of metabolite levels in *Arabidopsis thaliana* wild-type (WT) and *dapat* at the end of the night (EN).

**Supplementary Table 1.** Selected genes and primers used for expression analysis in *Arabidopsis* wild-type (WT) and *dapat* mutant plants submitted to GA_3_ treatment.

**Supplementary Figure S8**. Correlogram of significant (p < 0.05) Pearson correlations between metabolite levels and gene expression in *Arabidopsis* wild-type (WT) and *dapat* mutant plants at the end of the day.

**Supplementary Figure S9**. Correlogram of significant (p < 0.05) Pearson correlations between metabolite levels and gene expression in wild-type (WT) and *dapat* mutant plants at the end of the night.

1. **SUPPLEMENTARY DATA**

**
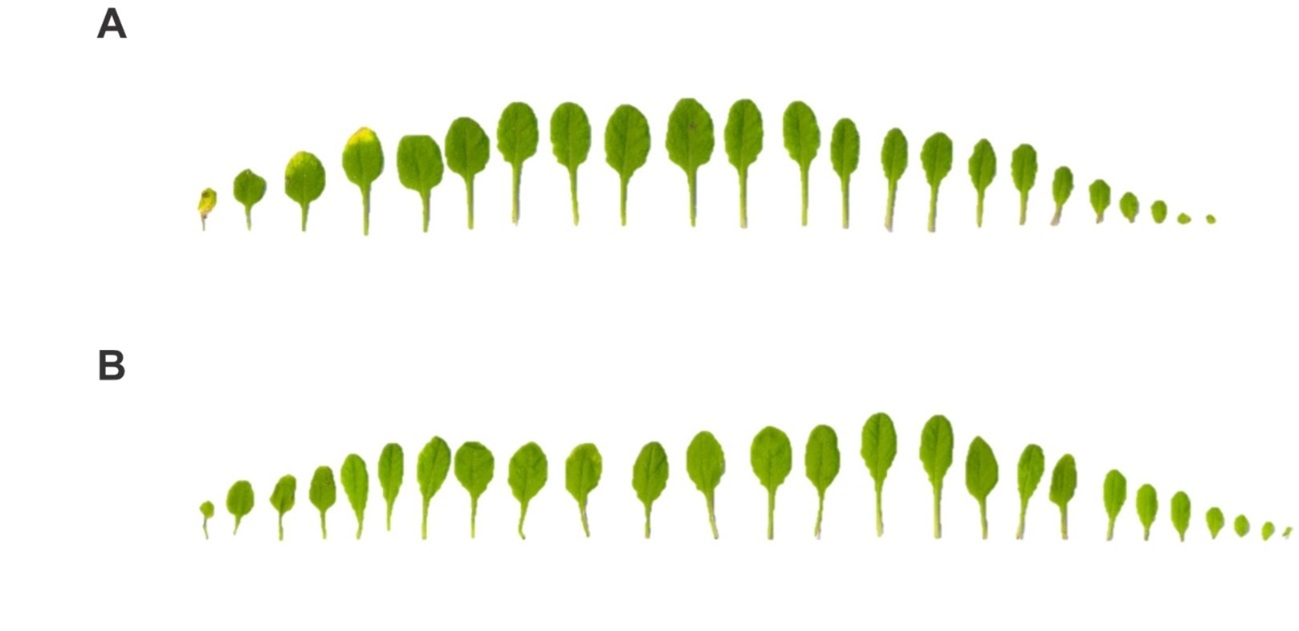
**

**Supplementary Figure S1. GA regime positively impacts the number of leaves in the *dapat* mutant plants.** Rosette leaf of 5–week–old *dapat* under different conditions. (Control) (A) or following GA_3_ treatment (+ GA) (B).

**
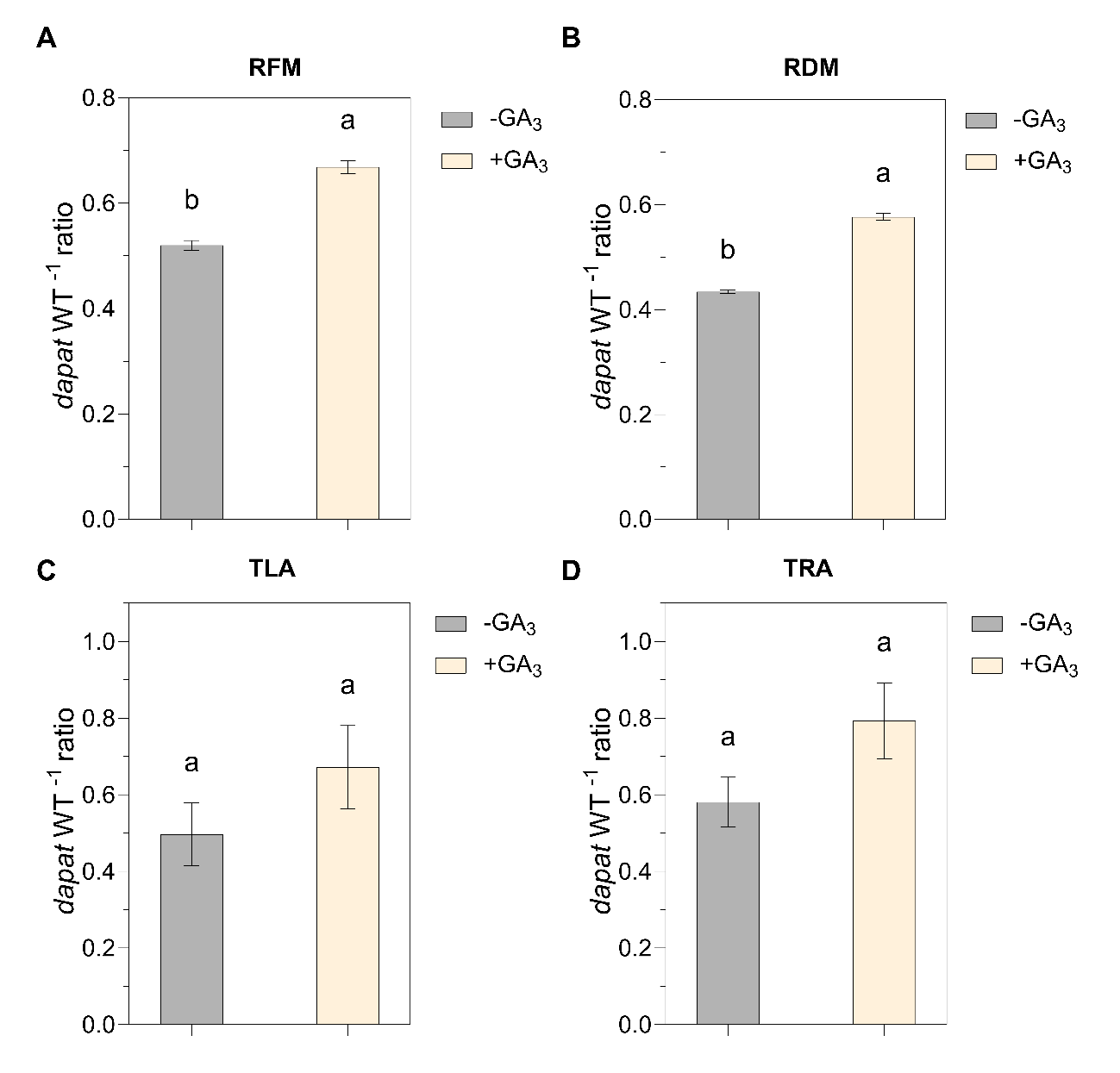
**

**Supplementary Figure S2. Gibberellin partially restores biomass allocation in the lysine-deficient *dapat* mutant plants.** Gibberellin (GA₃) application increases root biomass in the *Arabidopsis* lysine-deficient *dapat* mutant. Ratios of fresh weight (A), dry weight (B), total leaf area (C), and total rosette area (D). Error bars show SEM; different letters denote significant differences (P < 0.05).

**
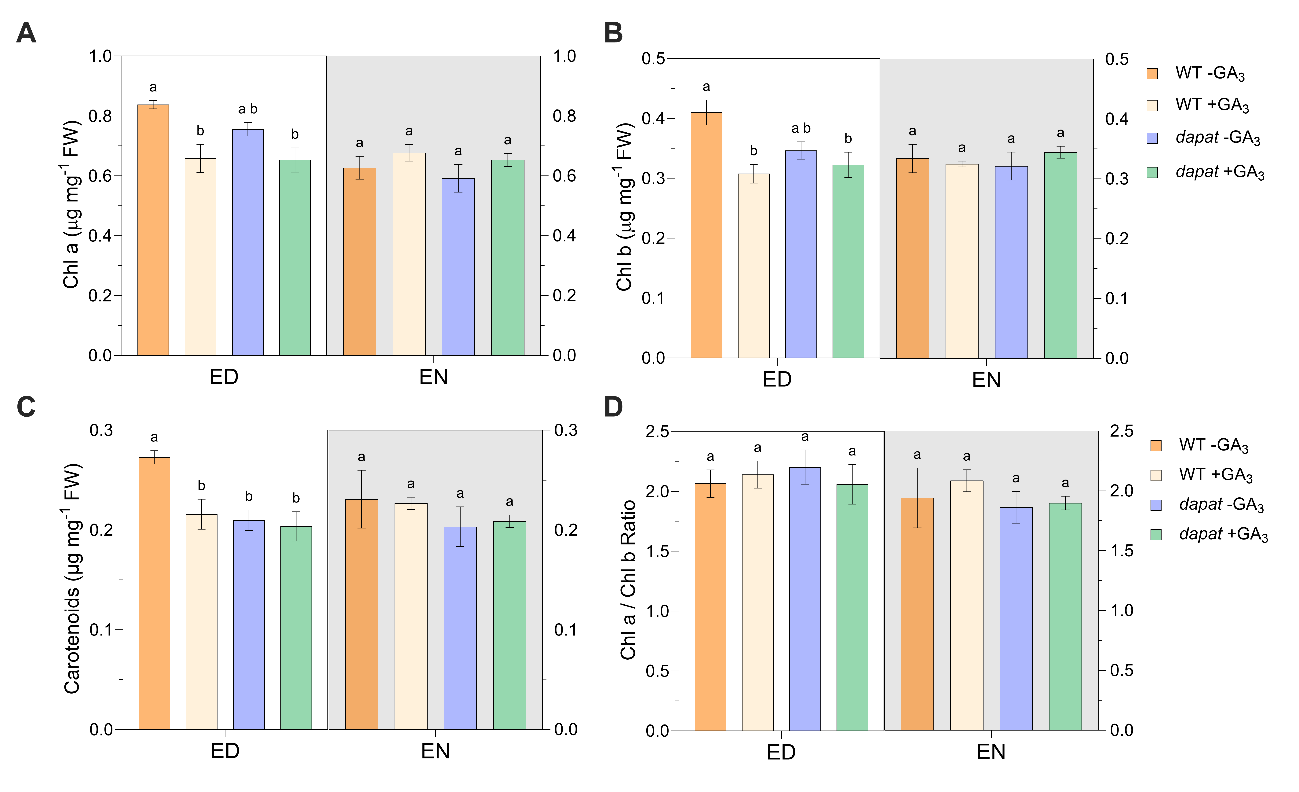
Supplementary Figure S3. GA treatment negatively impacts pigment content in both wild-type (WT) and *dapat* mutant plants.** Variation in chlorophylls and carotenoid contents in leaves of *Arabidopsis thaliana* under either control conditions (control) or following GA_3_ treatment (+GA). Chlorophyll a, chlorophyll b, carotenoids, and Chla/Chlb ratio were measured at the end of the day (A) and the end of the night (B). Two-way ANOVA, followed by Tukey's HSD test at 5% probability for mean comparison. Statistical tests were performed separately for the end of the day (ED) and end of the night (EN) time points. Values represent the mean ± standard error of five independent samples.

**Supplementary Table 1.** Selected genes and primers used for expression analysis in *Arabidopsis* wild-type (WT) and *dapat* mutant plants submitted to GA_3_ treatment.

| Gene | Locus | Forward Primer | Reverse Primer |  |
| --- | --- | --- | --- | --- |
| ***AGD2*** | AT4G33680 | CTGGAGAAGACTCATGTG | AGATGTTCTCTCTGTGACC |  |
| ***LKR/SDH*** | AT4G33150 | TGATTGTCGCGTCTCTGTATC | ATCTAGCCGAACTGCTTCTAC |  |
| ***D2HGDH*** | AT4G36400 | GAAGCTGTCATATCGGTGGA | TCGTACCCAGTATTGTCTTTGC |  |
| ***IVDH*** | AT3G45300 | AATGGGAAAGTTGACCCAAAGGAC | TAAAGCGACCTGCGTTGCTCTC |  |
| ***ETFQO*** | AT2G43400 | TTGGCCATTAGTGCTATGGAACAC | TCCCATGCTTGAGCGTGAAAGG |  |
| ***CPS*** | AT4G02780 | CTCAATGTCGCTAGAGAC | GTTGCCACTCATGTATTAG |  |
| ***KO*** | AT5G25900 | ATGTCTGAAGTCTCCACTC | AGGACCATAAATCTCTGAC |  |
| ***KS*** | AT1G79460 | GGTGGAGTACTTACAACG | CATGTTCTGAGCTGTACTC |  |
| ***GA3OX1*** | AT1G15550 | AGACGATCTCCTCTTCTC | AGTTCTACATGCATGACC |  |
| ***GA20OX1*** | AT4G25420 | CGGGACTACTTTAGAGAG | TCCTGTTCCTAGTGTGAG |  |
| ***TPS8*** | AT1G70290 | TTACCGCACATTGCCTGTTC | ATGCCTCGCGTAATCAAACG |  |
| ***DIN6*** | AT3G47340 | TGGCTTGTTCGACTGCAAAG | AATGCCACGTTCTTGCCATC |  |
| ***ATL8*** | AT1G76410 | TGCGGTTCTTCTTTGTGCAC | CTTTGTTGGCTGCAGCTACC |  |
| ***EF1a*** | AT5G60390 | CACGAGTCTCTTCTTGAG | TCATCCTTGGAGTTAGAG |  |
|  |  |  |  |  |

**
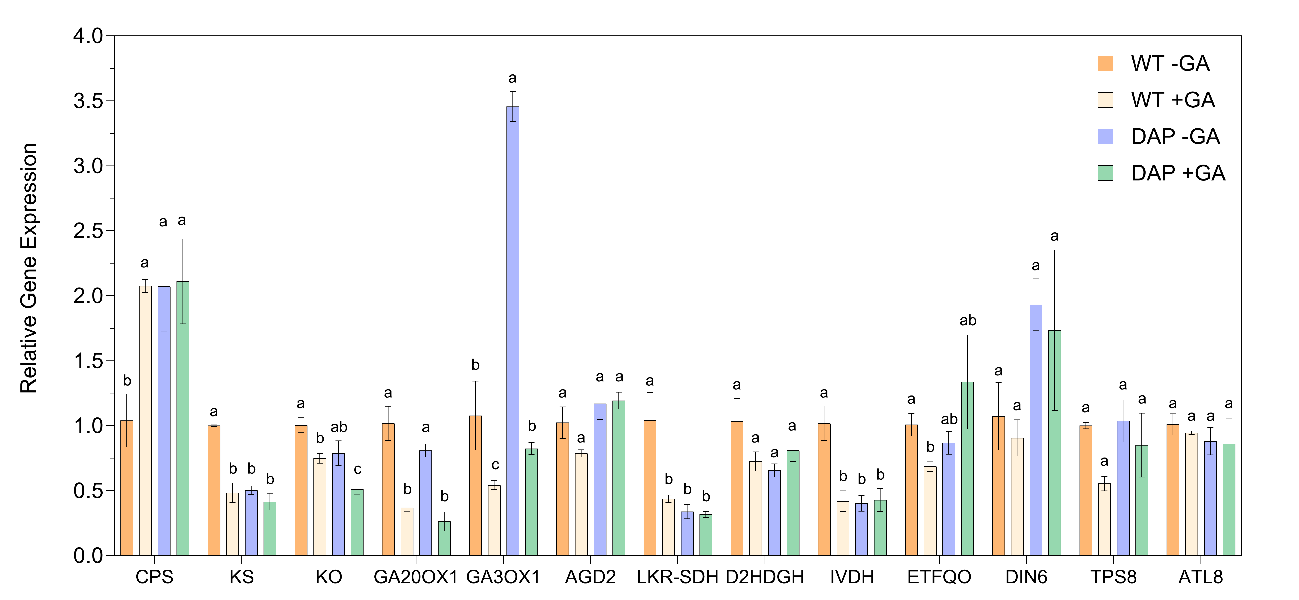
**

**Supplementary Figure S4.** Relative expression levels of genes encoding enzymes involved in GA and amino acids metabolism at the end of the day. *Arabidopsis* wild-type (WT) and *dapat* mutant plants grown under control conditions or following GA₃ treatment (+GA), with samples collected at the end of the day (ED). RT-qPCR analysis of transcript levels of *CPS* gene (At4g02780), *KS* (At1g79460), *KO* (At5g25900), *GA20ox1* (At4g25420), *GA3ox1* (At1g15550), *AGD2 (DAPAT* gene, At4g33680)*, LKR/SDH (*At4g33150)*, D2HGDH (*At4g36400)*, IVDH (*At3g45300)*, ETFQO (*At2g43400). Expression levels are normalized 2^-ΔCt^ at the end of the day (white background). Data are the mean of three replicates (± SEM).

**
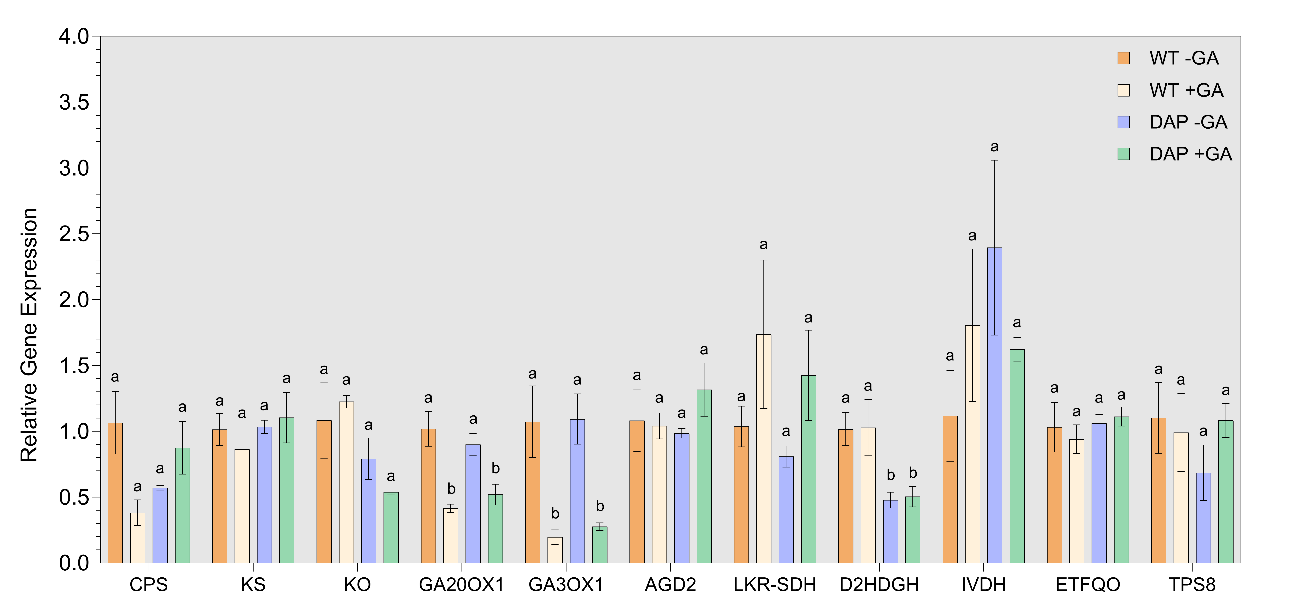
Supplementary Figure S5**. Relative expression levels of genes encoding enzymes involved in GA and amino acids metabolism at the end of the night. *Arabidopsis* wild-type (WT) and *dapat* mutant plants grown under control conditions or following GA₃ treatment (+GA), with samples collected at the end of the night (EN). RT-qPCR analysis of transcript levels of *CPS* gene (At4g02780), *KS* (At1g79460), *KO* (At5g25900), *GA20ox1* (At4g25420), *GA3ox1* (At1g15550), *AGD2 (DAPAT* gene, At4g33680)*, LKR/SDH (*At4g33150)*, D2HGDH (*At4g36400)*, IVDH (*At3g45300)*, ETFQO (*At2g43400). Expression levels are normalized 2^-ΔCt^ at the end of the night (gray background). Data are the mean of three replicates (± SEM).


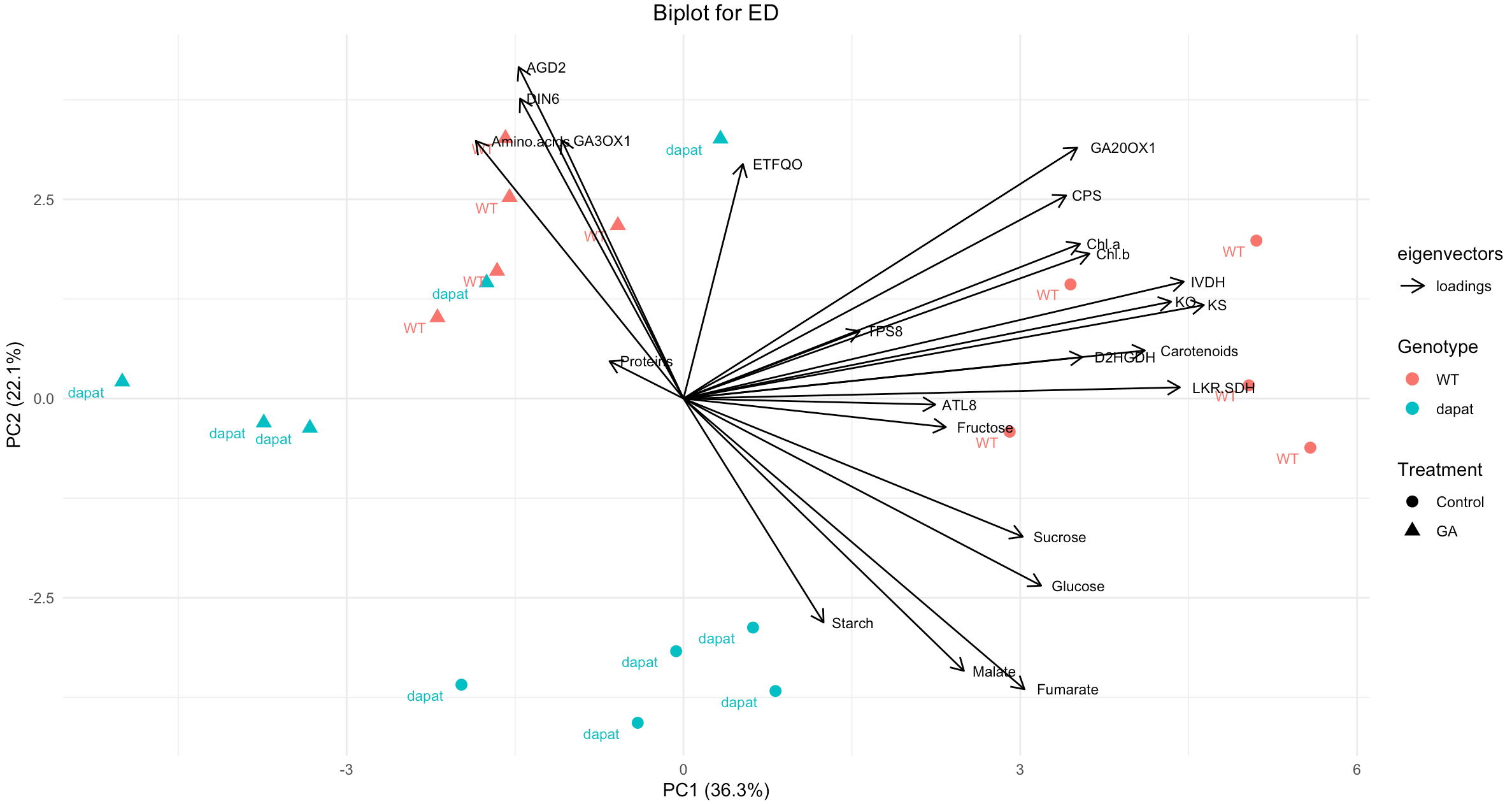


**Supplementary Figure S6**. Principal component analysis (PCA) of metabolite levels in *Arabidopsis thaliana* wild-type (WT) and *dapat* mutant plants at the end of the day (ED). Each data point represents an individual plant. Genotypes are represented by colors (WT = orange, *dapat* = cyan) and treatments are represented by symbols (Control: circle, GA: triangle). PC1 and PC2 are the first and second principal components, respectively, and the percentage of variance explained by each component is shown in parentheses. The variance explained by each PC and its component variables are highlighted in the accompanying table.


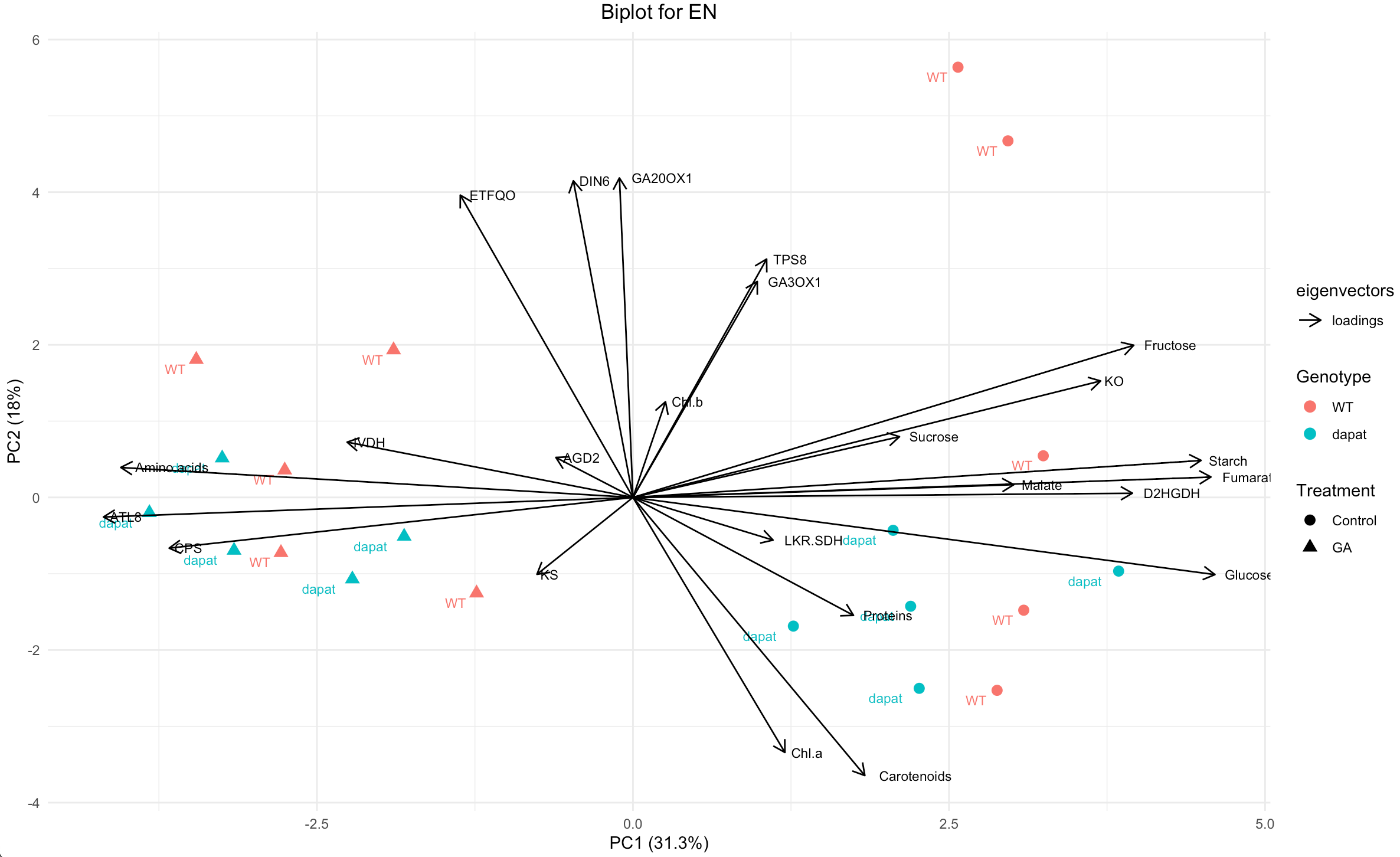

**Supplementary Figure S7**. Principal component analysis (PCA) of metabolite levels in *Arabidopsis thaliana* wild-type (WT) and *dapat* mutant plants at the end of the night (EN). Each data point represents an individual plant. Genotypes are represented by colors (WT = orange, *dapat* = cyan) and treatments are represented by symbols (Control: circle, GA: triangle). PC1 and PC2 are the first and second principal components, respectively, and the percentage of variance explained by each component is shown in parentheses. The variance explained by each PC and its component variables are highlighted in the accompanying table.


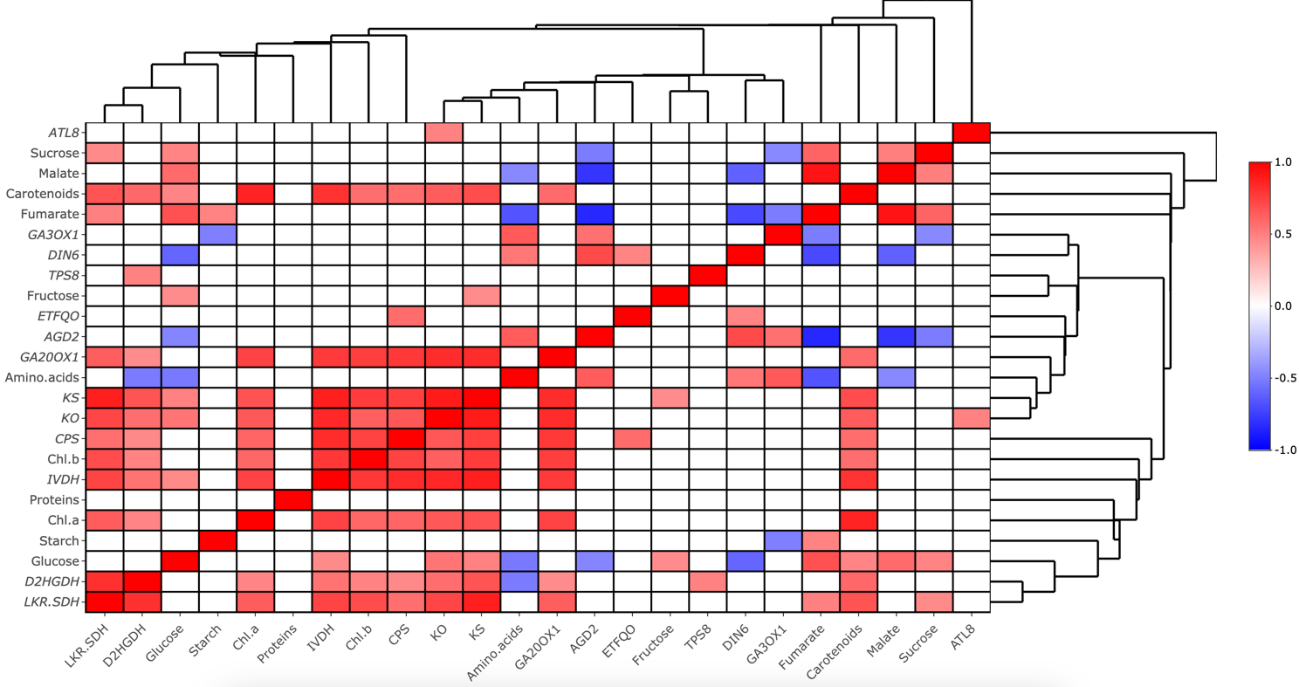


**Supplementary Figure S8**. Correlogram of significant (p < 0.05) Pearson correlations between metabolite levels and gene expression in *Arabidopsis* wild-type (WT) and *dapat* mutant plants at the end of the day. The correlogram was hierarchically clustered based on Pearson correlation coefficients. Shades of blue indicate negative correlations and shades of red indicate positive correlations. The intensity of the color represents the strength of the correlation.

**
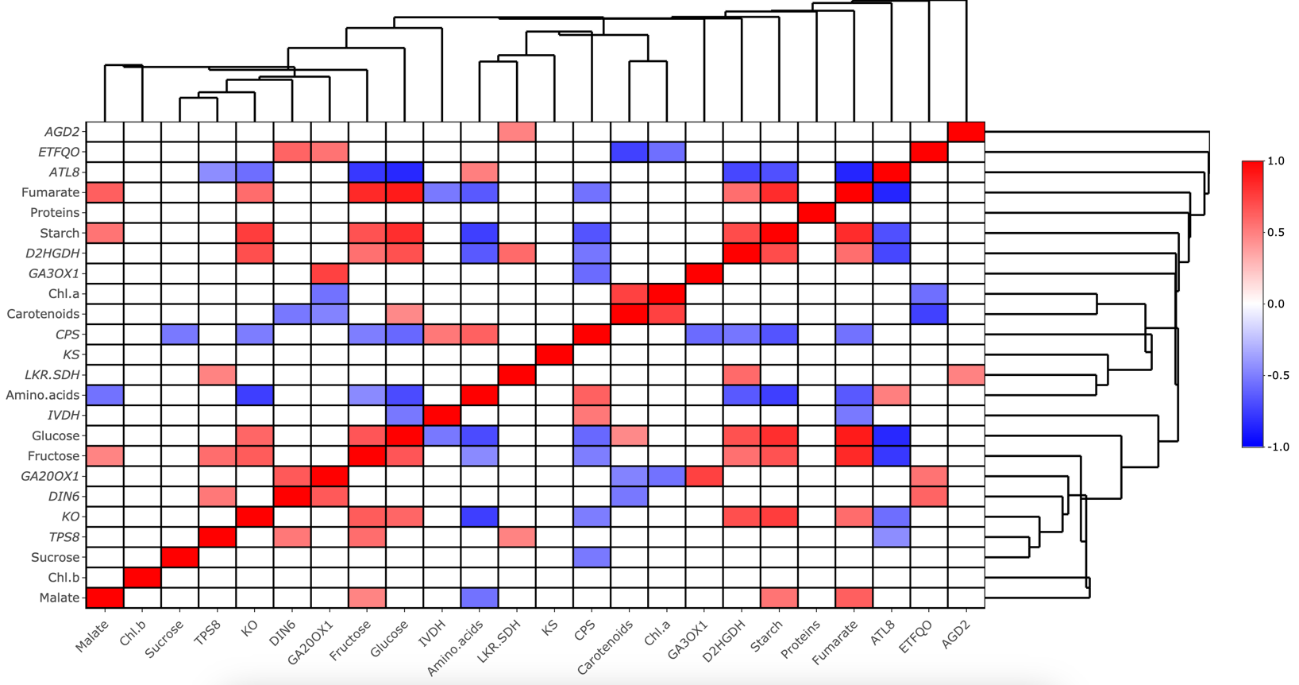
Supplementary Figure S9**. Correlogram of significant (p < 0.05) Pearson correlations between metabolite levels and gene expression in *Arabidopsis* wild-type (WT) and *dapat* mutant plants at the end of the night. The correlogram was hierarchically clustered based on Pearson correlation coefficients. Shades of blue indicate negative correlations and shades of red indicate positive correlations. The intensity of the color represents the strength of the correlation.
